# Impact of comprehensive lifestyle interventions on plasma branched-chain amino acid concentrations: a randomized trial

**DOI:** 10.1016/j.ajcnut.2025.10.008

**Published:** 2025-11-04

**Authors:** Yu Jin Lim, Rob M van Dam

**Affiliations:** 1 Department of Exercise and Nutrition Sciences, Milken Institute School of Public Health, The George Washington University, Washington, D.C., United States; 2 Saw Swee Hock School of Public Health, National University of Singapore, Republic of Singapore

**Keywords:** branched-chain amino acids, dietary patterns, weight loss, fitness, randomized controlled trial

## Abstract

**Background:**

Elevated plasma branched-chain amino acid (BCAA) concentrations are associated with a higher risk of type 2 diabetes and cardiovascular diseases. Lifestyle interventions have been proposed as a strategy to manage plasma BCAA concentrations, but evidence of their effectiveness is limited.

**Objectives:**

We investigated the effects of comprehensive lifestyle interventions on plasma BCAA concentrations over 6 mo and associations between changes in body mass index (BMI), physical fitness, and dietary factors and plasma BCAA changes.

**Methods:**

The PREMIER study was a randomized trial of the effects of behavioral lifestyle interventions. The interventions included counseling on diet, exercise, and weight loss (“Established”), a similar intervention with additional guidance to follow Dietary Approaches to Stop Hypertension (“Established plus DASH”), and an Advice-Only control group. We analyzed data from 713 male and female adult participants during the 6-mo intervention period. Data and biospecimens were obtained through the National Heart, Lung, and Blood Institute Biologic Specimen and Data Repository Information Coordinating Center repository, and plasma BCAA concentrations were measured using nuclear magnetic resonance spectroscopy. Multiple linear regression was used to assess the association between intervention groups and BCAA concentrations.

**Results:**

The Established [−7.19 μmol/L; 95% confidence interval (CI): 17.45, 3.08] and Established plus DASH (−8.70 μmol/L; 95% CI: −18.95, 1.55) interventions were associated with nonsignificant decreases in BCAA concentrations compared with the control group. Changes in BMI were correlated with changes in BCAA concentrations during the trial (partial Pearson *r* = 0.24, *P* < 0.001). Although changes in fitness and fiber intake were also significantly correlated with changes in BCAA concentrations, adjustment for BMI attenuated these correlations. Changes in the DASH and healthy plant-based diet indices and BCAA and protein intakes were not significantly correlated with plasma BCAA changes.

**Conclusions:**

Weight loss resulting from lifestyle interventions is associated with reductions in plasma BCAA concentrations. Improvements in fitness and diet composition are not associated with changes in BCAA concentrations independent of weight loss.

This trial was registered at clinicaltrials.gov as NCT0000616.

## Introduction

Branched-chain amino acids (BCAAs) include the essential amino acids isoleucine, leucine, and valine. BCAAs are known for their anabolic properties, promoting protein synthesis and inhibiting proteolysis, which is crucial for preventing muscle loss [[1], [2], [3], [4]]. In addition, BCAAs have been implicated in energy homeostasis and glucose metabolism [5]. In recent prospective studies, elevated BCAA concentrations were associated with a higher risk of type 2 diabetes and cardiovascular diseases (CVD) [[6], [7], [8], [9], [10]]. In a meta-analysis of 9 prospective nested case-control studies, elevated BCAA concentrations were associated with a higher risk of type 2 diabetes [9]. In the Women’s Health Study [10] and among individuals under 60 y in a meta-analysis of 9 prospective studies [7], higher BCAA concentrations were also associated with a higher CVD risk. Finally, a meta-analysis of genome-wide association studies showed an association between genetic variation in BCAA metabolism and type 2 diabetes, supporting a causal role of BCAAs in diabetes development [11].

The adverse health outcomes associated with elevated plasma BCAAs highlight the importance of strategies to manage BCAA concentrations. In cross-sectional studies in Singapore and the United States, higher physical activity levels were associated with lower BCAA concentrations [12,13]. In the United States Preventing Obesity Using Novel Dietary Strategies (POUNDS LOST) and the Israeli Dietary Intervention Randomized Controlled Trial (DIRECT), diet-induced weight loss was associated with reductions in plasma BCAAs [14]. A diet and exercise weight-loss intervention also reduced BCAA concentrations in German adolescents [15] but not in participants with prediabetes in the United States Diabetes Prevention Program [8]. In addition, adherence to a Mediterranean diet enriched with extra-virgin olive oil but not with nuts significantly reduced plasma BCAAs [16].

In this context, the PREMIER randomized trial offers an opportunity to evaluate the impact of lifestyle interventions on BCAA concentrations. This comprehensive trial assessed the effects of various behavioral lifestyle interventions, including weight loss, increases in physical activity, and following the Dietary Approaches to Stop Hypertension (DASH) diet [17,18]. Our analysis of the PREMIER trial aims to elucidate how different lifestyle interventions affect plasma BCAA concentrations over a 6-mo period. We also evaluated correlations between changes in BMI, fitness, and dietary factors [i.e., intakes of BCAA, protein, and fiber, the healthy Plant-based Dietary Index (hPDI) and the DASH index] and plasma BCAAs.

## Methods

The PREMIER trial was a multicenter randomized controlled trial designed to assess the impact of lifestyle interventions on blood pressure and other cardiovascular disease risk factors [17,18]. We obtained data and plasma samples from this trial through the National Heart, Lung, and Blood Institute Biologic Specimen and Data Repository Information Coordinating Center repository and measured metabolites, including BCAAs, in the plasma samples. A total of 810 participants were recruited between September 1999 and June 2001 [17,18]. We used data from the baseline and the 6-mo visit for the current analysis. The study protocol was approved by the institutional review board at each of the 4 participating clinical centers (Johns Hopkins University in Baltimore, MD; Pennington Biomedical Research Center in Baton Rouge, LA; Duke University Medical Center in Durham, NC; and Kaiser Permanente Center for Health Research in Portland, OR). The current data analysis was based on deidentified data and was exempted from Institutional Review Board review by the George Washington University Office of Human Research. All participants provided written informed consent.

### Study population

Participants included in this study were 25 y or older with a BMI between 18.5 and 45.0 kg/m^2^ [17]. Individuals were excluded if they had a history of cardiovascular events or were receiving antihypertensive drugs or other medications affecting blood pressure. Individuals with a history of psychological or emotional problems who had been hospitalized for such issues within the past 2 y were excluded. Participants undergoing treatment for serious illnesses such as cancer, HIV, or liver or kidney disease were also excluded unless they had been in remission for over 2 y and had not required further treatment within the past 2 y. In addition, females who were pregnant, planning to become pregnant within the next 2 y, or breastfeeding were excluded. Participants were also excluded if they had excessive alcohol consumption, took diet pills or any medications to control weight, or if their weight changed >15 lbs within 3 mo before the screening. Physical limitations that could hinder moderate-intensity physical activity were also grounds for exclusion, as were physician-imposed restrictions on physical activity. Finally, participants could not have plans to leave the area within the next 2 y or reside in the same household as a PREMIER staff member or another PREMIER participant.

### Interventions

Participants were randomly assigned to 1 of 3 intervention groups: “Advice-Only,” “Established,” or “Established + DASH.” A computer program was used to centrally conduct randomization assignments stratified by clinic and hypertension status, with a block size of 24, aiming for a balanced assignment to the 3 intervention arms [17,18]. The clinical center staff subsequently notified participants of the intervention to which they were assigned. Staff involved in follow-up data collection were blinded to participant treatment assignments, and the staff involved in the interventions were blinded to all outcome data.

The Advice-Only group received recommendations for weight loss for those who were overweight, limiting intake of alcohol and sodium, engaging in regular physical activity, and consuming a healthful diet low in fat and cholesterol. The recommendations were provided at the beginning of the study, and the participants received printed educational materials. The Established and Established plus DASH interventions followed the same schedule of 14 group meetings and 4 individual counseling sessions during the first 6 mo. For the Established intervention, the goals included reducing weight by ≥7 kg (15 lbs.) if overweight, limiting sodium intake, reducing fat intake to 30% or less of total caloric intake, engaging in 180 min/wk of moderate-intensity physical activity, and limiting alcohol intake to ≤1 ounce of ethanol/d for males and 0.5 ounces for females. The Established plus DASH group had the same goals as the Established group for weight loss, physical activity, lowering salt intake, and restricting alcohol consumption. In addition, the intervention emphasized the DASH dietary pattern with high consumption of fruit and vegetables (9–12 servings/d) and low-fat dairy (2–3 servings/d) and reducing saturated (≤7% of calories) and total fat (≤25% of calories) intakes.

### Measurements

The clinical staff was unaware of participants' intervention assignments during the trial. Measurements of weight, fitness, and dietary intake were conducted at baseline and after 6 mo. Fitness was assessed using a submaximal treadmill test, which measured heart rate response to a sex- and age-specific workload. A lower heart rate at the set workload indicated improved fitness. Because increased physical activity enhances cardiorespiratory fitness, fitness changes were used as a marker for changes in physical activity levels. Weight was recorded with participants wearing light clothing and no shoes, using a calibrated scale. Height was measured once at baseline with a wall-mounted stadiometer. BMI was calculated by dividing weight (kg) by height squared (m^2^). Dietary intake was assessed using 2 unannounced 24-h dietary recalls conducted via telephone at each assessment point. Baseline data on age, sex, race, marital status, education, and smoking history were collected through standardized questionnaires [17,18].

All dietary variables were calculated using the mean of the 2 dietary recalls at both baseline and 6 mo. To assess the healthfulness of the overall dietary pattern, we calculated scores based on adherence to the hPDI [19] and DASH [20] diets. The original scores were developed for cohort studies and based on quintiles. We slightly changed the scoring to make the scores more sensitive to changes in intakes in the context of a trial. Specifically, for healthy foods, we assigned a score of 10 for intakes higher than the 90th percentile (rounded to 0.5) of baseline intakes and a 0 for having no intake. For unhealthy foods, we assigned a score of 10 for having no intake and a score of 0 for intakes higher than the 90th percentile. Scores between 0 and 10 were assigned proportionally. For the DASH score, the Pearson correlation between the used DASH score and the conventional DASH score was 0.80 at baseline and 0.81 at the 6-mo visit. For the hPDI score, the correlation between the used hPDI and conventional hPDI was 0.59 at baseline and 0.63 at the 6-mo visit. For hPDI, we categorized foods into 18 groups that were classified into 3 categories: healthy plant foods, less healthy plant foods, and animal foods. For the healthy plant food, no intake was given the lowest score, and intakes higher than the 90th percentile were given the maximum score (3.5 servings for coffee and tea, 1.5 for fruits, 1 for legumes, 0.5 for nuts and seeds, 4 for vegetables, 1.5 for vegetable oils, and 3 for wholegrains). For the animal food and less healthy plant food groups, no intakes were given the highest score, whereas intakes higher than the 90th percentile were given the minimum score (90th percentile: 2 daily servings for animal fat, 4.5 for dairy, 1 for eggs, 1.5 for fish, 3.5 for meat, 4 for miscellaneous animal products, 1 for fruit juice, 2 for potatoes, 7 for refined grains, 2 for sugar-sweetened beverages, and 6.5 for sweets and desserts).

The DASH score was assigned based on the DASH score criteria [20]. Food intakes were categorized into 7 categories. For fruit, vegetables, nuts and legumes, whole grains, and low-fat dairy intakes higher than the 90th percentile were given the maximum score, whereas no intake was given the lowest score (4.5 for fruits, 2 for low-fat dairy, 2 for nuts and legumes, 5 for vegetables, and 4 for whole grains). For red and processed meat and sweetened beverages, no intakes were given the highest score, whereas intakes higher than the 90th percentile were given the minimum score (90th percentile: 3 daily servings for red and processed meat, 2 for sweetened beverages).

Fasting blood samples were collected to measure the plasma BCAA concentration. Plasma BCAA concentrations (i.e., valine, isoleucine, and leucine) were measured using nuclear magnetic resonance spectroscopy by Nightingale Health Ltd [21].

### Statistical analysis

The sample size of the PREMIER study was determined based on the original aim to determine the impact of lifestyle interventions on systolic blood pressure [17]. The primary outcome of interest for the current analysis was the change in total plasma BCAA concentrations, and secondary outcomes were the change in specific BCAA concentrations (i.e., leucine, isoleucine, and valine).

Multiple linear regression models were used to compare plasma BCAA changes between the intervention groups using the Advice-Only control group as the reference. We adjusted for the following covariates to reduce residual variance due to outcome predictors and to control for chance imbalances between the intervention groups: age (years), sex (male or female), region (4 study sites), race (African American or other), education level (less than high school, college, or graduate school), marital status (single, married, divorced/separated, or widowed), cigarette smoking status (never, past, or current), alcohol consumption (g/d), and baseline BCAA concentrations (μmol/L). Statistical tests for this regression model indicated no evidence of heteroskedasticity (*P* = 0.68). Furthermore, the *Q*–*Q* plot of residuals showed no substantial deviation from normality, and the Cook’s distance plot did not identify any influential outliers that would substantially affect the results. A separate analysis was performed with stratification by race and sex.

As an exploratory analysis, we examined changes in BMI, fitness, and dietary factors during the intervention in relation to changes in BCAA concentrations. Specifically, we calculated Pearson correlation coefficients for changes in BMI (kg/m^2^), fitness (heart rate in beats/min), fiber intake (g/1000 kcal), hPDI score, DASH score, BCAA intake (% kcal), protein intake (% kcal), and changes in BCAA concentrations. These correlations were adjusted for potential confounding by age, sex, region, race, education level, marital status, cigarette smoking, and alcohol consumption. In addition, we examined whether BMI may explain the correlations for fitness and dietary factors by further adjusting for BMI.

We also conducted mediation analyses to estimate the extent to which changes in BMI mediated the impact of the interventions on BCAA concentrations. This analysis was based on a counterfactual-based framework implemented in the Stata medeff package [22,23]. Linear regression models were used to estimate the direct and indirect effects of the interventions on BCAA concentrations in the presence of BMI changes as a mediator. Two multivariable regression models were specified: 1 for BCAA outcomes (as a function of the interventions, BMI changes, and covariates) and 1 for BMI changes (as a function of the interventions and covariates). Each model was adjusted for the same covariates as used for the main intervention analysis. For each invention (i.e., Established and Established plus DASH), the intervention's total effect, direct effect, indirect effect (i.e., mediation by BMI changes), and the percentage of total effect mediated were estimated based on 5000 Monte Carlo simulations and 1000 Bootstrap replications.

The main analysis comparing randomized groups was based on 713 participants. We conducted complete case analyses for the correlation and mediation analyses of BMI (*N =* 705) and the correlation analyses for fitness (*N =* 635) and dietary factors (*N =* 659). Statistical significance was defined as a *P* value < 0.05, with 2-tailed tests used throughout the analysis. Statistical analyses were conducted using STATA version 18 (StataCorp).

## Results

Of the 810 participants of the PREMIER trial, 3 participants were excluded based on missing data for smoking or marital status, and 94 based on a lack of availability of blood samples or valid BCAA measurements at baseline or the 6-mo follow-up visit (Supplemental Figure 1). As a result, the current analysis included 713 participants.

The average age of participants was 50.18 (SD 8.91) y, and the average BMI was 32.96 kg/m^2^ (SD 5.68). Most participants were females (61%) and married (66%), and about one-third (33%) identified as African American. Furthermore, most participants had received higher education and never smoked. Participants were assigned to 3 intervention groups: Advice-Only, Established, and Established plus DASH. Although most factors were consistent among the 3 intervention groups, the Advice-Only group had a slightly higher proportion of participants who were females and African American, and a lower proportion of past smokers, and alcohol consumers than the Established plus DASH group (Table 1). The Advice-Only group also had lower plasma BCAA concentrations than the Established plus DASH group.

**TABLE 1:** Baseline participant characteristics by lifestyle intervention group.

Characteristics	Total (*N* = 713)	Advice-Only (*N* = 242)	Established (*N* = 232)	Established+DASH (*N* = 239)
Age (y)	50.18 (8.91)	49.80 (8.84)	50.14 (8.66)	50.61 (9.24)
Sex				
Male	277 (39)	91 (38)	82 (35)	104 (44)
Female	436 (61)	151 (62)	150 (65)	135 (56)
Race				
African American	238 (33)	88 (36)	83 (36)	67 (28)
Other	475 (67)	154 (64)	149 (63)	172 (72)
Marital status				
Single	83 (12)	25 (10)	29 (12)	29 (12)
Married	475 (66)	157 (65)	157 (68)	161 (67)
Divorced/separated	121 (17)	46 (19)	42 (18)	33 (14)
Widowed	34 (5)	14 (6)	4 (2)	16 (7)
Education level				
High school or less	60 (8)	15 (6)	17 (7)	28 (12)
College	420 (59)	156 (65)	134 (58)	130 (54)
Graduate school	233 (33)	71 (29)	81 (35)	81 (34)
Cigarette smoking				
Never	452 (63)	158 (65)	153 (66)	141 (59)
Past	231 (33)	76 (32)	64 (28)	91 (38)
Current	30 (4)	8 (3)	15 (6)	7 (3)
Alcohol consumer	346 (49)	115 (48)	103 (44)	128 (54)
BMI (kg/m^2^)	32.96 (5.68)	32.73 (5.51)	33.04 (5.46)	33.11 (6.06)
Exercise heart rate (beats/min)	130.16 (14.41)	129.96 (14.43)	130.30 (14.41)	130.22 (14.46)
Energy intake (kcal)	1917.13 (623)	1885.61 (608)	1915.61 (625)	1950.53 (636)
Protein intake (% kcal)	16.08 (4.12)	15.78 (3.93)	16.34 (4.62)	16.13 (3.77)
BCAA intake (% kcal)	2.75 (0.72)	2.70 (0.69)	2.79 (0.78)	2.77 (0.68)
Fiber intake (g/1000 kcal)	9.17 (3.72)	9.25 (3.78)	9.20 (3.77)	9.07 (3.63)
hPDI score	79.54 (14.52)	80.02 (15.06)	79.04 (13.36)	79.54 (15.09)
DASH score	30.71 (11.62)	31.29 (12.31)	30.07 (11.23)	30.75 (11.28)
Plasma BCAA (μmol/L)	449.27 (74.44)	442.72 (72.32)	446.85 (77.02)	458.25 (73.44)

Data are numbers (%) or means (SD).

Abbreviations: BCAA, branched-chain amino acids; DASH, Dietary Approaches to Stop Hypertension; hPDI, healthy Plant-based Diet Index.

Table 2 shows the changes in lifestyle-related variables and BCAA concentrations according to the intervention groups. Reductions in BMI and exercise heart rate during the trial were greater in the treatment groups compared with the control group. Increases in fiber and protein intake and the DASH score were most pronounced in the Established plus DASH group. In contrast, increases in the hPDI score were the most pronounced in the Established group. The changes in BCAA intake were similar for the 3 groups. The associations between intervention groups and changes in plasma BCAA concentrations are shown in Supplemental Table 1 (crude) and Table 3 (multivariable-adjusted). In the crude model, the Established intervention reduced BCAA concentrations by 9.30 μmol/L [95% confidence interval (CI): −21.51, 2.91] and the Established plus DASH intervention by 15.93 μmol/L (95% CI: −28.05, −3.81). After multivariable adjustment, these associations were weaker and not statistically significant for either the Established (−7.19; 95% CI: 17.45, 3.08) and Established plus DASH (−8.70; 95% CI: −18.95, 1.55) intervention. This attenuation of the associations after multivariable adjustment was mainly due to adjustment for baseline BCAA concentrations. The associations were similar for the individual BCAAs. We also conducted multivariable-adjusted analyses stratified by race and sex. The Established plus DASH intervention was associated with significant reductions in total BCAA, isoleucine, and leucine concentrations in participants who were not African American (predominantly White), but not in those who were African American (Supplemental Table 1). However, the *P* values for interaction between this intervention and race were not significant (total BCAA: 0.17, isoleucine: 0.17, leucine: 0.10). In addition, none of the other interactions between race and sex and the interventions were significant.

**TABLE 2:** Change in BMI, fitness, and diet during the trial by intervention group.

	Total (*N =* 713)	Advice-Only (*N =* 242)	Established (*N =* 232)	Established +DASH (*N =* 239)
BMI (kg/m^2^)	−1.39 (1.78)	−0.48 (1.09)	−1.75 (1.88)	−1.96 (1.88)
Exercise heart rate (beats/min)	−7.38 (10.65)	−4.94 (9.43)	−8.22 (11.22)	−8.98 (10.85)
Fiber intake (g/1000 kcal)	2.39 (4.47)	0.94 (4.12)	2.07 (4.16)	4.14 (4.51)
Protein intake (% kcal)	1.35 (5.05)	1.10 (5.02)	1.00 (5.25)	1.92 (4.84)
BCAA intake (% kcal)	−1.20 (0.83)	−1.22 (0.78)	−1.21 (0.91)	−1.16 (0.78)
hPDI score	2.07 (16.61)	−0.25 (15.99)	4.44 (15.88)	2.11 (17.61)
DASH score	6.08 (11.67)	0.94 (9.93)	5.92 (10.99)	11.31 (11.63)

Data are means (SD).

Abbreviations: BCAA, branched-chain amino acids; DASH, Dietary Approaches to Stop Hypertension; hPDI, healthy Plant-based Diet Index.

**TABLE 3:** Differences in plasma BCAA concentration changes for the Established and Established plus DASH lifestyle interventions compared with the control group in 713 participants.

Intervention	Model	BCAA		Isoleucine		Leucine		Valine	
		Beta	95% CI	Beta	95% CI	Beta	95% CI	Beta	95% CI
Established	Crude	−9.30	−21.51, 2.91	−0.06	−2.61, 2.50	−4.48	−9.66, 0.71	−5.80	−12.15, 0.54
	Multivariable	−7.19	−17.45, 3.08	−0.08	−2.22, 2.07	−2.68	−6.63, 1.28	−4.86	−10.35, 0.63
Established +DASH	Crude	−15.93	−28.05, −3.81	−2.97	−5.51, −0.43	−7.76	−12.91, −2.61	−6.43	−12.73, −0.13
	Multivariable	−8.70	−18.95, 1.55	−1.72	−3.86, 0.42	−3.74	−7.69, 0.21	−3.44	−8.91, 2.03

Betas (95% CI) represent the regression coefficient (95% confidence interval) for the effect of the intervention on plasma BCAA, isoleucine, leucine, and valine (μmol/L) compared with the reference group (Advice-Only).

Crude refers to the unadjusted model

Multivariable refers to the model with adjustment for age, sex, region, race, education, marital status, smoking status, baseline alcohol consumption, and baseline concentrations of total or individual BCAA.

Abbreviations: BCAA, branched-chain amino acids; DASH, Dietary Approaches to Stop Hypertension.

We also examined correlations between changes in BMI, fitness, and dietary factors and changes in plasma BCAAs. We calculated unadjusted (Supplemental Figure 2) and partial (Table 4) Pearson correlation coefficients. Increases in BMI were significantly associated with increases in concentrations of total BCAAs (partial *r* = 0.24, *P* < 0.001) and all individual BCAAs. The increase in exercise heart rate was also associated with higher BCAA concentrations (*r* = 0.15, *P* < 0.001). Because increases in exercise heart rate reflect decreases in participants’ fitness, these results indicate that reduced fitness was associated with increased BCAA concentrations. In addition, increases in fiber intake were significantly associated with decreases in valine concentrations (*r* = −0.11, *P* = 0.006). However, after adjusting for BMI changes, the associations of exercise heart rate and fiber intake with BCAA concentrations were attenuated and no longer significant (Table 4). Changes in hPDI score, DASH score, BCAA intake, and protein intake were not significantly associated with changes in BCAAs, either before or after adjustment for BMI.

**TABLE 4:** Pearson partial correlations between changes in lifestyle factors and changes in plasma BCAA concentrations during the trial.

	BCAA		Isoleucine		Leucine		Valine	
	*r*	*P* value	*r*	*P* value	*r*	*P* value	*r*	*P* value
BMI	0.24	< 0.001	0.23	< 0.001	0.18	< 0.001	0.22	< 0.001
Exercise heart rate	0.15	< 0.001	0.12	0.003	0.10	0.01	0.13	0.001
BMI-adjusted	0.07	0.10	0.04	0.35	0.04	0.28	0.05	0.20
Fiber intake	−0.08	0.06	−0.02	0.65	−0.02	0.63	−0.11	0.006
BMI-adjusted	−0.04	0.37	0.03	0.51	0.01	0.80	−0.07	0.07
hPDI score	−0.02	0.65	−0.003	0.94	0.01	0.77	−0.03	0.43
BMI-adjusted	−0.01	0.78	0.005	0.90	0.02	0.67	−0.02	0.54
DASH score	−0.01	0.83	0.002	0.95	0.02	0.68	−0.03	0.51
BMI-adjusted	0.03	0.41	0.05	0.25	0.05	0.25	0.01	0.75
BCAA intake	0.01	0.73	0.03	0.50	0.01	0.72	−0.0002	1.00
BMI-adjusted	0.03	0.40	0.05	0.23	0.03	0.50	0.02	0.65
Protein intake	0.002	0.97	0.01	0.71	−0.002	0.97	0.001	0.98
BMI-adjusted	0.03	0.48	0.04	0.28	0.02	0.69	0.03	0.50

Pearson correlation coefficients were adjusted for age, sex, region, race, education, marital status, smoking status, alcohol consumption, and baseline concentrations of total or individual BCAA.

The analyses were based on 705 participants for BMI, 635 participants for fitness, and 659 participants for dietary factors.

The units were kg/m^2^ for BMI, g/1000 kcal for fiber intake, beats/min for exercise heart rate, percent of energy intake for BCAA and protein intakes, and μmol/L for BCAA concentrations.

Abbreviations: BCAA, branched-chain amino acids; DASH, Dietary Approaches to Stop Hypertension; hPDI, healthy Plant-based Diet Index.

Given the strong correlation between changes in BMI and changes in plasma BCAAs, we examined the extent to which changes in BMI may mediate the effect of the interventions on BCAA concentrations (Supplemental Table 2). The estimated indirect effect, reflecting mediation by BMI, was significantly associated with changes in BCAA concentrations for both the Established and Established plus DASH interventions. In contrast, the estimated direct effects of the interventions on BCAA concentrations, which reflect effects independent of BMI, were small and nonsignificant. The estimated percentages mediated suggest nearly complete mediation of the intervention effects on BCAA concentrations by BMI changes.

## Discussion

We conducted a longitudinal analysis of the PREMIER trial comparing the effects of different lifestyle interventions on plasma BCAA concentrations. Both the Established and Established plus DASH groups received comprehensive lifestyle interventions focused on diet, physical activity, and weight loss. The Established plus DASH group additionally emphasized the DASH dietary pattern. Plasma BCAAs decreased significantly for the Established plus DASH group compared with the control group, although these changes were not statistically significant after multivariable adjustment. Our findings indicate that weight loss was the major determinant of reductions in BCAA concentrations in the context of these lifestyle interventions. BMI changes were strongly correlated with changes in BCAA concentrations during the trial and largely explained the effects of the interventions on BCAA concentrations.

Increases in fiber intake were correlated with reductions in valine concentrations, and improvements in fitness were correlated with reductions in all BCAA concentrations; however, these correlations were largely explained by changes in BMI. Furthermore, changes in diet quality as reflected by DASH or hPDI scores and protein or BCAA intakes were not significantly associated with changes in BCAA concentrations. In line with these findings, we did not observe a substantial difference in the impact of the Established plus DASH and the Established intervention on BCAA concentrations. The findings of this study support the growing evidence that lifestyle interventions accompanying weight loss are associated with reductions in plasma BCAAs. In the POUNDS LOST and DIRECT trials, weight loss diets significantly reduced plasma BCAAs in individuals with obesity [14]. Similarly, a controlled trial that included an intensive behavioral weight loss phase followed by a weight maintenance phase found that plasma BCAA decreased as weight loss occurred [24]. However, the results of the Diabetes Prevention Program trial in persons with impaired glucose tolerance present a contradictory perspective. Although participants in the lifestyle intervention group achieved significant weight loss [25], the intervention did not significantly reduce plasma BCAAs compared with the control group [8]. This finding raises the possibility that lifestyle weight-loss interventions reduce BCAA concentrations less in people with impaired glucose tolerance.

The impact of adiposity on plasma BCAAs is likely due to metabolic disruptions in individuals with higher adiposity. BCAA concentrations are elevated in obesity due to impaired catabolism in the muscle, leading to higher circulating BCAAs [26]. This has been linked to insulin resistance and metabolic dysfunction [27]. Exercise heart rate was positively associated with BCAA concentrations in our study, indicating that enhanced physical fitness resulting from physical activity may contribute to a reduction of plasma BCAA. Muscle metabolic health, which could lead to increased utilization of BCAA, potentially lowers plasma BCAA concentrations [28,29]. In addition, physical activity can promote branched-chain aminotransferase, which catalyzes the transamination of BCAAs in skeletal muscle [30,31]. However, the attenuation of the exercise heart rate association with BCAA concentrations after adjusting for BMI suggests that the effect of physical activity on weight loss at least partly mediates the benefits of physical activity on BCAA concentrations.

A key strength of our study was the use of a randomized controlled trial design. We also examined the longitudinal association between changes in specific lifestyle factors and changes in BCAA concentrations. However, despite adjustments for a range of potential confounding factors, residual confounding by other behavioral changes may have influenced the results of these analyses. In our mediation analysis, some estimates of the percent mediation of the interventions’ effect by BMI exceeded 100%. This result reflects inconsistent mediation where the direct and indirect effects have opposite signs [32]. Inconsistent mediation may result from the opposite effects of weight loss and other aspects of the intervention on BCAA levels, model misspecifications such as unmeasured confounding, or sampling error [32,33]. Thus, the reported percentage mediation should be interpreted with caution, and focusing on the direction and magnitude of the estimated indirect effects is more appropriate. Another limitation of our study was that dietary intakes were self-reported for only a few days. In addition, the assessment of fitness may not have captured all relevant aspects of physical activity, such as exercise intensity. These sources of measurement error likely introduced nondifferential misclassification, potentially weakening the observed associations for dietary factors and physical activity, as reflected in fitness. Therefore, future studies using alternative methods to assess diet (e.g., biomarkers) and physical activity (e.g., accelerometers) are warranted. Additionally, the 6-mo duration of the study may limit the understanding of the long-term effects of lifestyle changes on plasma BCAAs. Finally, the study population was mostly White, with approximately one-third comprising African American adults. We observed a significant effect of the Established plus DASH intervention on BCAA levels in White participants but not in African American participants, although the interaction was not statistically significant. Thus, further research is needed to determine whether the impact of lifestyle interventions on BCAA levels may differ between racial and ethnic groups.

In conclusion, we did not find a significant effect of lifestyle counseling on improving physical activity and dietary quality in the PREMIER trial. However, our analysis suggests that lifestyle interventions can reduce plasma BCAA concentrations when accompanied by substantial weight loss. Increases in fitness and fiber intake were associated with reductions in BCAA concentrations in our analysis, but this appeared to be explained by weight loss. Nevertheless, our results do not exclude the possibility that larger or longer-term changes in specific dietary factors or physical activity behaviors can independently reduce BCAA concentrations. Further research is also needed to explore the biological mechanisms underlying the effects of adiposity on BCAA concentrations.

## Author contributions

The authors’ responsibilities were as follows – RMvD: designed research; YJL: analyzed data; and all authors: interpreted results, wrote the paper, had primary responsibility for final content, and read and approved the final manuscript.

## Data availability

Deidentified participant data described in the manuscript and the code book will be made available on request pending application and approval from the National Heart, Lung, and Blood Institute Biologic Specimen and Data Repository Information Coordinating Center**.** The analytical code can be provided on request from the corresponding author.

## Declaration of generative AI and AI-assisted technologies in the writing process

During the preparation of this work, the authors used GRAMMARLY to improve readability and language. After using this, the authors reviewed and edited the content as needed and take full responsibility for the publication’s content.

## Funding

This manuscript was prepared using PREMIER research materials obtained from the NHLBI Biologic Specimen and Data Repository Information Coordinating Center (BioLINCC). We thank the PREMIER participants, staff, and researchers for their contributions to this research. Research reported in this publication was supported by the NHLBI of the NIH under Award Number R21HL169803.

The content is solely the responsibility of the authors and does not necessarily represent the official views of the PREMIER study or the NIH.

## Conflict of interest

RMvD reports financial support was provided by National Heart Lung and Blood Institute (NHLBI). If there are other authors, they declare that they have no known competing financial interests or personal relationships that could have appeared to influence the work reported in this paper.
