# Supplementary material for: Impact of comprehensive lifestyle interventions on plasma branched-chain amino acid concentrations: a randomized trial
Source: Am J Clin Nutr. 2025 Nov 4;122(6):1829–35. doi: 10.1016/j.ajcnut.2025.10.008 (PMC12799384; doi:10.1016/j.ajcnut.2025.10.008)

**Supplementary Materials**

Title: Impact of Comprehensive Lifestyle Interventions on Plasma Branched-Chain Amino Acid Concentrations: A Randomized Trial

First author: Yu Jin Lim

**Supplemental Table 1.** Differences in plasma BCAA concentration changes for the Established and Established plus DASH lifestyle interventions compared with the Advice-only control group stratified by race and sex.

|  | | BCAA | | Isoleucine | | Leucine | | Valine | |
| --- | --- | --- | --- | --- | --- | --- | --- | --- | --- |
|  | | Beta | 95% CI | Beta | 95% CI | Beta | 95% CI | Beta | 95% CI |
| **Stratified by race** | |  |  |  |  |  |  |  |  |
| Established | African American | 0.30 | -16.55, 17.15 | 0.86 | -2.84, 4.55 | -1.77 | -8.83, 5.29 | -1.56 | -10.41, 7.30 |
|  | Other | -12.52 | -25.47, 0.42 | -0.83 | -3.45, 1.78 | -3.61 | -8.38, 1.15 | -7.30 | -14.29, -0.31 |
|  | P interaction | 0.39 |  | 0.69 |  | 0.82 |  | 0.44 |  |
| Established + DASH | African American | 2.33 | -15.75, 20.40 | 0.56 | -3.40, 4.53 | 1.13 | -6.43, 8.70 | -1.87 | -11.36, 7.62 |
|  | Other | -14.80 | -27.32, -2.29 | -3.00 | -5.52, -0.47 | -6.04 | -10.65, -1.43 | -4.66 | -11.41, 2.09 |
|  | P interaction | 0.17 |  | 0.17 |  | 0.10 |  | 0.78 |  |
| **Stratified by sex** | |  |  |  |  |  |  |  |  |
| Established | Male | -9.91 | -27.76, 7.93 | -1.05 | -5.02, 2.92 | -3.16 | -10.25, 3.94 | -8.03 | -18.00, 1.94 |
|  | Female | -5.92 | -18.87, 7.02 | 0.36 | -2.22, 2.94 | -2.58 | -7.43, 2.27 | -3.32 | -10.02, 3.37 |
|  | P interaction | 0.73 |  | 0.43 |  | 0.88 |  | 0.46 |  |
| Established + DASH | Male | -10.48 | -27.19, 6.23 | -2.59 | -6.30, 1.12 | -4.85 | -11.48, 1.79 | -4.32 | -13.67, 5.02 |
|  | Female | -6.37 | -19.73, 6.99 | -0.80 | -3.47, 1.86 | -2.44 | -7.45, 2.58 | -2.09 | -8.98, 4.81 |
|  | P interaction | 0.64 |  | 0.33 |  | 0.48 |  | 0.54 |  |

Betas are derived from linear regression analysis of the effect of the intervention on plasma BCAA.

Adjusted for potential confounders, including age, sex, region, race, education, marital status, smoking status, baseline alcohol consumption, and baseline concentrations of total or individual BCAA

Abbreviations: BCAA, Branched-Chain Amino Acid; CI, confidence interval; DASH, Dietary Approaches to Stop Hypertension.

**Supplementary Table 2.** Mediation analyses of the extent to which changes in BMI mediated the effects of the Established and Established plus DASH interventions compared to Advice only on plasma BCAA concentrations.

|  | | BCAA | | Isoleucine | | Leucine | | Valine | |
| --- | --- | --- | --- | --- | --- | --- | --- | --- | --- |
| Exposure | | Beta | 95% CI | Beta | 95% CI | Beta | 95% CI | Beta | 95% CI |
| Established | |  |  |  |  |  |  |  |  |
|  | Total effect | -8.10 | (-18.70, 2.53) | -0.23 | (-2.45, 2.02) | -3.14 | (-7.33, 1.04) | -5.16 | (-10.82, 0.48) |
|  | Direct effect | 1.05 | (-10.43, 12.58) | 1.92 | (-0.50, 4.35) | -0.42 | (-5.00, 4.17) | -0.74 | (-6.86, 5.41) |
|  | Indirect effect* | -9.15 | (-14.27, -4.51) | -2.15 | (-3.26, -1.15) | -2.71 | (-4.67, -0.93) | -4.42 | (-7.11, -1.98) |
|  | % Mediated | 113 | (106. 120) | 938 | (874, 1002) | 86 | (79, 94) | 86 | (82, 89) |
| Established + DASH | |  |  |  |  |  |  |  |  |
|  | Total effect | -9.65 | (-19.94, 0.77) | -1.73 | (-3.89, 0.46) | -3.78 | (-7.78, 0.26) | -4.22 | (-9.78, 1.38) |
|  | Direct effect | 3.94 | (-7.13, 15.06) | 0.93 | (-1.41, 3.29) | -0.02 | (-4.43, 4.41) | 2.92 | (-3.06, 8.93) |
|  | Indirect effect* | -13.60 | (-19.07, -8.52) | -2.66 | (-3.82, -1.60) | -3.76 | (-5.80, -1.88) | -7.14 | (-10.10, -4.40) |
|  | % Mediated | 141 | (140, 142) | 154 | (151, 157) | 99 | (98, 101) | 169 | (169, 170) |

*Indirect effect refers to the estimated effect that is mediated by BMI.

The beta (95% CI) estimates are from linear regression analyses within a causal mediation framework with 5,000 Monte Carlo simulations and 1,000 Bootstrap replications adjusted for age, sex, region, race, education, marital status, smoking status, alcohol consumption, and baseline concentrations of total or individual BCAAs.

The analyses were based on 705 participants without missing values for BMI.

Abbreviations: BMI, body mass index; BCAA, Branched-Chain Amino Acid; DASH, Dietary Approaches to Stop Hypertension; CI, confidence interval.

**Supplementary Figure 1.** Flow diagram of the selection of the analytical population.

**Supplementary Figure 2**. A Heat Map based on unadjusted Pearson correlation coefficients between changes in lifestyle variables, BMI, and branched-chain amino acid (BCAA) concentrations during 6 months of the PREMIER trial. Fitness refers to the exercise heart rate. Abbreviations: DASH, Dietary Approaches to Stop Hypertension; BCAA, Branched-Chain Amino Acid; BMI, Body Mass Index; hPDI, healthy Plant-based Diet Index.


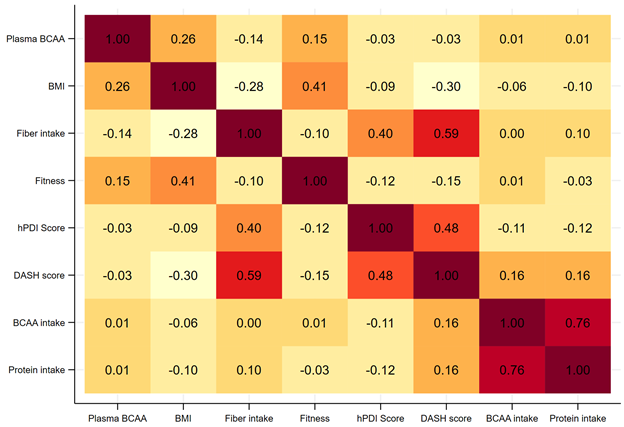

Supplement: multimedia component 1 [file mmc1.docx]
